# Contrasting trait-based assembly mechanisms on different trophic levels: ants and plants on wood-pastures

**DOI:** 10.1007/s00442-025-05802-4

**Published:** 2025-09-30

**Authors:** Ádám Lőrincz, Kata Frei, Alida Anna Hábenczyus, Bonita Ratkai, Gábor Lőrinczi, András Kelemen, Csaba Tölgyesi, Zoltán Bátori, István Elek Maák

**Affiliations:** 1 Department of Ecology, University of Szeged, Szeged, 6726 Hungary; 2 Doctoral School in Biology, University of Szeged, Szeged, 6726 Hungary; 3 Doctoral School of Environmental Sciences, University of Szeged, Szeged, 6720 Hungary; 4 MTA-SZTE ‘Momentum’ Applied Ecology Research Group, University of Szeged, Szeged, 6726 Hungary; 5 Momentum’ Seed Ecology Research Group, Institute of Ecology and Botany, HUN-REN Centre for Ecological Research, Vácrátót, 2163 Hungary; 6 Museum and Institute of Zoology, Polish Academy of Sciences, 01-818 Warsaw, Poland

**Keywords:** Community organization, Interspecific interactions, Environmental filtering, Functional ecology, Heterogeneity

## Abstract

Complex landscapes (i.e., those harboring multiple habitat types at immediate spatial proximity) are highly relevant to both applied and theoretical ecological research, yet the mechanisms shaping functional trait distributions and diversity metrics across trophic levels in these systems remain poorly understood. To address this knowledge gap, we used wood-pastures as a model system and focused on two prominent groups occupying different trophic levels: plants and ants. We sampled three Central-European wood-pastures, with all four encompassed habitat types (grasslands, solitary trees, forest edges, and forests; 48 sites in total). Our results revealed significant differences in taxonomic and functional composition for both groups among the different habitat types of wood-pastures. However, the underlying mechanisms driving these patterns differed between plants and ants. Based on RLQ and fourth-corner analyses, heterogeneity in environmental conditions mainly influenced plant functional trait distributions. In contrast, ant diversity metrics and trait distributions were strongly linked to vegetation characteristics and habitat structure, and were only indirectly influenced by local microclimate, as shown by path analyses. These highlight that while mapping the increased environmental heterogeneity of complex landscapes, the main mechanisms shaping functional composition and diversity metrics might differ for organisms at different trophic levels (i.e., predominantly environmental filtering for plants and interspecific competition for ants). Consequently, the patterns and peaks of taxonomic and functional diversity do not necessarily coincide for different organisms in complex landscapes, emphasizing that conservation initiatives should focus on the system as a whole, rather than individual habitat types, to maximize biodiversity conservation.

**Supplementary Information:**

The online version contains supplementary material available at 10.1007/s00442-025-05802-4.

## Introduction

According to the ‘habitat heterogeneity hypothesis’ originally proposed by MacArthur and MacArthur (1961), spatial heterogeneity in abiotic and biotic conditions promotes coexistence and, therefore, leads to increased species richness by expanding the number of partitionable niche dimensions. Arguably one of the most striking pieces of evidence supporting this hypothesis is that heterogeneous habitat features (e.g., scattered trees in open landscapes), geomorphologic structures (e.g., topographic depressions in karst landscapes), or landscapes (e.g., forest-grassland mosaics) often harbor disproportionally high biodiversity compared to their spatial extent (Manning et al. 2006; Kelemen et al. 2017; Bátori et al. 2023). Recognizing their potential conservation value, multiple studies focused on understanding the drivers governing diversity patterns in similar heterogeneous habitats (e.g., Kiebacher et al. 2017; Erdős et al. 2018; Frei et al. 2023). Moreover, by promoting high spatial and temporal environmental heterogeneity within relatively small areas, such habitats often serve as model systems for studying community assembly processes at small- and meso-scales, with important implications both in theoretical and applied contexts (Chytrý et al. 2023; Deák et al. 2024; Lőrincz et al. 2024a,b).

Despite this growing research interest, substantial knowledge gaps remain in understanding community organization in heterogeneous landscapes, largely due to the lack of two interacting elements: trait-based studies and multitrophic approaches. Our understanding is limited regarding how and to what extent the different abiotic and biotic conditions influence functional trait distributions (i.e., the spatial distribution of trait values or attributes) of organisms in landscapes where multiple habitat types/microhabitats co-occur at immediate spatial proximity (hereafter: complex landscapes). Local community structure and composition is primarily shaped by the interactive effects of abiotic, biotic, and dispersal filtering mechanisms (Götzenberger et al. 2012; Cadotte and Tucker 2017), which affect the occurrences and abundances of organisms based on their functional traits. Given that complex landscapes, such as forest-grassland mosaics or many silvopastoral systems, harbor multiple habitat types within small spatial scales (e.g., closed forests, forest patches of different sizes, forest edges, solitary trees, and grasslands), they might mimic large-scale environmental gradients by promoting heterogeneity in key environmental parameters (Lőrincz et al. 2024a; Ho et al. 2024). As such, communities at different habitat types are expected to show substantial dissimilarities in their functional trait compositions, thereby increasing landscape-level functional diversity.

To truly explore the capacity of complex landscapes to shape functional trait distributions and enhance landscape-level functional diversity, an integrative approach that includes taxa from different trophic levels is needed. Multitrophic approaches provide a more comprehensive understanding of how biodiversity is related to ecosystem functioning and stability (Schuldt et al. 2018; Seibold et al. 2019). Moreover, these approaches can also help to identify recurring patterns in community assembly processes, thereby revealing taxon-independent general structuring mechanisms (Frenette-Dussault et al. 2013). However, while recent research efforts have been aimed at identifying the factors shaping functional characteristics in heterogeneous habitats, most studies have focused almost exclusively on plants (Ottaviani et al. 2019; Deák et al. 2024; Frei et al. 2025), whereas taxa from higher trophic levels are rarely considered (but see Bátori et al. 2020, 2022).

**Fig. 1:**
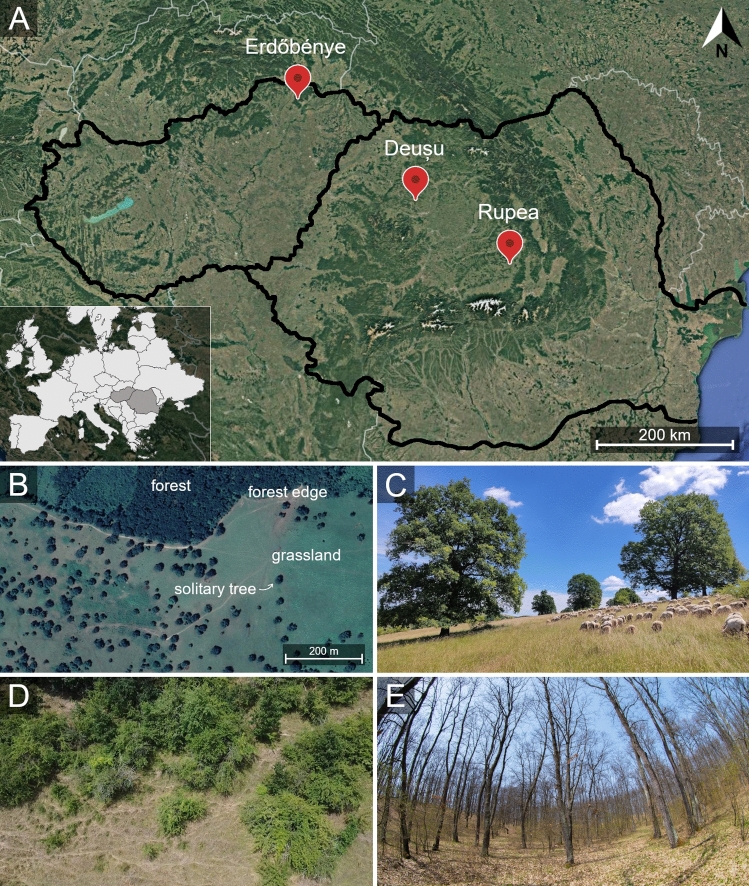
**A** Location of the sampled wood-pastures in the Carpathian Basin, Central Europe (Hungary and Romania), **B** Aerial view of a wood-pasture (Rupea, Romania), with the four different habitat types, **C** solitary trees embedded in a grassland matrix, **D** forest edge, **E** forest

In this study, we aimed to bridge these knowledge gaps by addressing the direct and indirect factors, as well as possible cascading mechanisms shaping functional trait composition and functional diversity of communities across different trophic levels in complex landscapes. To achieve this, we used wood-pastures as a model system and focused on the communities of two ecologically prominent and diverse groups occupying different trophic levels: plants and ants. Wood-pastures are ancient silvopastoral systems, which, in a broader sense, are viewed as complex landscapes (Bergmeier et al. 2010), as they accommodate four different habitat types on a relatively small scale (ca. 100 m): grasslands, solitary trees, forest edges, and forests (Fig. 1B–E). Each habitat type has been shown to provide unique micro-environmental conditions and to influence the taxonomic composition of local communities (Gallé et al. 2017; Tölgyesi et al. 2018), thus providing a valuable system to reveal the factors influencing trait-environment relationships and different aspects of community organization in complex landscapes (Garbarino and Bergmeier 2014; Hartel et al. 2014; Lőrincz et al. 2024a,b). We chose ants to represent higher trophic levels in our study, as their sheer abundance, dominance, and various ecological functions make them a fundamental component of most terrestrial ecosystems (Schultheiss et al. 2022). Ants are reliable and widely used indicators of local site conditions (Andersen et al. 2002), as they are highly sensitive and responsive to changes in habitat conditions (Perfecto and Vandermeer 1996). Moreover, by forming direct (e.g., nesting in plants) and indirect (e.g., trophobiosis with sap-sucking hemipterans) relationships with plants, the community attributes of ants are often linked to vegetation properties (Nooten et al. 2019; Brassard et al. 2023). We specifically hypothesized that (I) the contrasting abiotic and biotic conditions across different habitat types will select for species with different traits, leading to significant differences in both the taxonomic and functional composition of plant and ant communities. We assume that (II) at least some of this variation is directly linked to microclimate and expect significant associations between species trait values/attributes and environmental conditions for both groups. However, (III) we expect that the strength and nature of these relationships will vary over time, becoming stronger in warm seasons, when the environmental contrast between the different habitat types is greater. Finally, given the direct and indirect relationships between plants and ants, we (IV) expect strong bottom-up effects on ant diversity metrics (i.e., taxonomic and functional diversity).

## Materials and methods

### Study sites

We carried out our study on three wood-pastures located along a 390 km transect within the Carpathian Basin, Central Europe (Fig. 1A). The wood-pastures selected for our study are comparable in size and share similar management regimes and environmental characteristics (Table S1). Solitary trees–the characteristic features of wood-pastures, embedded in a grazed grassland matrix–are mostly oaks (*Quercus petraea* and *Q. robur*), but hornbeam (*Carpinus betulus*) and fruit trees, mainly pear (*Pyrus pyraster* and *P. communis*) are also present (Hartel et al. 2018). The grassland matrix is grazed year-round by sheep, cattle, and buffalo. Each wood-pasture is nested in a hilly landscape and connected to second-growth oak-hornbeam forests of various sizes, forming forest edges with the grasslands.

### Data collection

Following Lőrincz et al. (2024a,b), data collection–including vegetation and ant sampling, along with local microclimate measurements–was conducted at fixed sampling sites within each habitat type (solitary trees, grassland matrix, forest edge, secondary forest interior) in 2022, making 48 sites in total (four habitat types × four spatial repetitions × three wood-pastures). We selected sampling sites with comparable physiognomies (e.g., grasslands without substantial shrub encroachment; solitary trees with similar undergrowth; forest and forest edge sites with comparable canopy openness). In woody habitat types (forests, forest edges, and solitary trees), each sampling site contained a central oak tree, which were spaced at least 20 m from one another, and at least 50 m from the nearest sampling site established in a different habitat type. In grasslands, the sampling sites were arranged in a rectangular pattern and were located at least 50 m from the nearest solitary tree or forest edge. The 50 m distance among plots of different habitat types was chosen to avoid any potential edge effects or cross-habitat effects (Ewers and Didham 2006). To assess whether seasonal changes in climatic conditions and vegetation structure influence the obtained patterns, we repeated the field sampling in spring (April) and summer (June). The sampling of the different habitat types of a wood-pasture was performed simultaneously, within one sampling day, under clear weather conditions. The sampling of different wood-pastures was conducted within a 10-day window in each season.

#### Vegetation sampling

To assess plant composition at the different habitat types, we randomly placed one 1 m × 1 m plot in each sampling site (i.e., 16 vegetation plots/wood-pasture; 48 in total). Plots of similar size have been shown to adequately capture within-habitat-type heterogeneity in wood-pastures (e.g., Tárrega et al. 2009; Tölgyesi et al. 2018). Plots under tree canopies were placed 2–3 m from the tree trunk. All vascular species (herbs, shrubs, and tree saplings) in the plots were identified to the species level and assigned a percentage cover value. In addition to vegetation cover, we also evaluated the percentage cover of leaf litter and bare ground in each plot.

#### Sampling of ant communities

We assessed ant species’ abundances and community composition at the different habitat types using non-invasive baiting. We placed baits, consisting of a teaspoon of a 1:3 tuna:honey mixture, on 8-cm diameter plastic disks at five different points within each sampling site (i.e., 80 baits/wood-pasture; 240 in total): four baits were placed 3 m from the central oak tree in the cardinal directions, while an additional bait was placed on the north-facing side of the tree’s trunk, at a height of 1.5 m to detect foraging ant workers on trees. In grasslands, the central bait was placed on the ground. We recorded the number of workers of each ant species on the baits, as well as aggressive interactions among them (e.g., biting, expulsion) at 30-min intervals during three observation periods: 7:30 a.m.–9:30 a.m., 11:30 a.m.–2:00 p.m., and 4:00 p.m.–6:30 p.m. (i.e., 17 observations × 240 baits; 4080 observations in total). Based on our previous works, the selected observation periods cover the main activity periods of diurnal species (Maák et al. 2020). Ants were identified to morphospecies or genus level in the field, and workers were collected and preserved in 95% ethanol after each observation period for later species identification relying on the keys of Czechowski et al. (2012), and Seifert (2018). The collected specimens were deposited at the Department of Ecology, University of Szeged.

#### Microclimate measurements

To link species occurrences to local microclimatic conditions, we monitored the fine-scale changes of five key environmental parameters, each regarded as a primary factor influencing plant and ant distributions at local and regional scales: soil moisture, soil and air temperature, relative air humidity, and solar irradiation (Perfecto and Vandermeer 1996; Barry 2008; De Frenne et al. 2013; Seifert 2017). Soil moisture (volumetric water content–VWC%) was measured in the upper 12 cm of soil at four locations (facing the cardinal directions) within each sampling site using a Fieldscout TDR 350 Soil Moisture Meter. We recorded soil temperature (°C) in the upper 10 cm of soil at the beginning of each ant observation period at each sampling site using digital penetration probe thermometers (TFA pocket-digitemp 30.1018). To record air temperature (°C) and relative air humidity (%), we installed two microclimate loggers (Optin ADL-TH3) at each sampling site, recording data at 5-min intervals. One logger, housed in a radiation shield, was suspended 10 cm above the ground, while the other was mounted to the south-facing side of the central oak tree’s trunk at breast height to measure microclimate experienced by ants foraging on the tree. We chose this side to avoid interference with the device while observing ant activity on arboreal baits. We measured solar irradiance (W/m^2^) at one location per habitat type using Kipp & Zonen SMP3 pyranometers. The sensors, recording data at 5-min intervals, were mounted to tripod stands and placed at least 20 m from the nearest sampling site to prevent shading by the observers.

#### Functional traits

##### Plant functional traits

We selected five plant functional traits to assess the functional characteristics of the plant communities. These were plant height, specific leaf area (SLA), leaf area (LA), start of flowering, and life form. Plant height and leaf traits (SLA and LA) are among the most important and ecologically most relevant traits as they are associated with principal biological functions: plant height reflects competitive ability, while SLA and LA indicate the rate of resource investment (Westoby 1998; Díaz et al. 2004). Leaf traits also correlate with palatable biomass for herbivores (Wilson et al. 1999). Floral traits, such as start of flowering, provide information about the reproduction strategy of plants (Kremen et al. 2007), while life forms can be used as a proxy to describe the general appearances of communities (Raunkiaer 1934). We collected the trait data from regional data sources (**Table S2**). For traits with multiple records, we calculated average trait values. We did not use trait data from databases for the height of tree saplings and shrubs as they refer to adult individuals and not the actual sampled woody saplings, thus we maximized plant height for shrubs and tree saplings at 120 cm.

##### Ant functional traits

We used six functional traits to characterize the ant communities: cephalic size, queen number, dominance, temperature and humidity optimum, and ecological plasticity. As certain ant functional traits can be habitat- and context-dependent (Punttila et al. 1996; Philpott et al. 2010), we relied on direct measurements and observations in relevant cases (e.g., dominance, or temperature and humidity optima; Table S2). Cephalic size is commonly used as a proxy for body size in ants (Seifert 2002), which constrains where workers are able to forage and is generally associated with multiple life-history traits (Chown and Gaston 2010). Queen number is linked to the reproductive strategy of ant colonies, as well as to the dispersal and colony founding strategies of young queens (Heinze and Foitzik 2009). Dominance, in simple terms, is the capability of a species to exclude others from resources and generally has two components in ants: behavioral (i.e., aggressiveness) and ecological (i.e., abundance) dominance (Parr and Gibb 2010). Ant communities are often organized in dominance hierarchies, where species at lower levels yield to stronger competitors (Savolainen and Vepsäläinen 1988). Air temperature and relative air humidity are primary environmental factors influencing ant foraging patterns, a crucial behavior for acquiring the nutrients necessary for growth, survival, and reproduction (Roeder et al. 2022). Finally, plasticity refers to the extent of variation in ecological, environmental, and habitat conditions that a given species is able to tolerate (Czechowski et al. 2012).

### Statistical analyses

First, we assessed whether the different habitat types of wood-pastures (grasslands, solitary trees, forest edges, and forests) influenced the taxonomic and functional composition of plant and ant communities. To achieve this, we calculated pairwise permutational multivariate analysis of variance (PERMANOVA) based on Bray–Curtis dissimilarity and 999 permutations, separately for plants and ants and each season. To compare taxonomic composition, we used the square-root transformed species × abundance data. For functional composition, we calculated community-weighted means (CWMs) at each sampling site for quantitative traits (plants: height, SLA, LA, and flowering start; ants: cephalic size, temperature and humidity optimum), while we evaluated the nominal traits (plants: life form; ants: dominance, queen number, and plasticity) by analyzing the plant cover and ant worker numbers of each level separately. We then prepared sample × trait matrices, with the values representing the CWMs for each trait at each sampling site (cf. Tölgyesi et al. 2019), and calculated distance matrices using Gower distance. For this, levels of the nominal traits were handled as dummy variables with fuzzy coding, and as the range of CWM values differed considerably, we min–max normalized them before analyses. The *p* values of PERMANOVAs were adjusted for multiple comparisons with the FDR method. To visualize the differences in functional composition among habitat types, we performed functional principal coordinate analyses (PCoAs) using the previously calculated distance matrices.

To assess the associations between functional trait values/attributes and environmental variables and to evaluate their strength, we employed the integrative approach of Dray et al. (2014), which combines two complementary analyses: the RLQ and fourth-corner methods (Dolédec et al. 1996; Legendre et al. 1997). As before, separate analyses were carried out for plants and ants during spring and summer. Prior to analyses, we organized our data into three tables: (1) Table R–environmental variables (mean values of plot-scale solar irradiation, air and soil temperature, relative air humidity, soil moisture, and cover of bare ground and litter), (2) Table L–species abundance data of plants and species incidence data of ants, and (3) Table Q–functional trait values for each species. The RLQ method integrates three separate analyses on R, L, and Q tables to identify the main relationships between environmental gradients and trait syndromes, mediated by species abundances. We applied correspondence analysis (CA) to Table L, principal component analysis (PCA) to Table R (after scaling the environmental variables), and Hill and Smith analysis to Table Q as it contained a mix of quantitative and qualitative variables. Both PCA and Hill and Smith analyses were constrained by species abundances. We assessed the overall significance of these relationships with multivariate tests on the total inertia of the RLQ analyses based on 999 permutations (model 2: environmental variables, R; model 4: species traits, Q). To support these ordinations statistically and test for bivariate associations between functional traits and environmental variables, we performed fourth-corner analyses based on 4999 permutations. The obtained *p* values were adjusted for multiple comparisons with the FDR method.

In addition to testing for direct trait-environment associations, we also assessed how local-scale habitat characteristics, vegetation, and microclimate influence ant diversity metrics via direct and indirect pathways. To achieve this, we performed path analysis by applying a piecewise structural equation modeling approach (SEM; Lefcheck 2016). To quantify local-scale habitat characteristics, we used two variables associated with soil surface complexity, each playing a major role in shaping community composition and diversity metrics of ground-dwelling ants: leaf litter- and bare ground cover (Bestelmeyer and Wiens 1996). For microclimate, we considered the mean values and ranges of each measured parameter (air and soil temperature, air humidity, solar irradiation, and soil moisture). However, as both the ground cover and microclimate variables were strongly correlated (Table S3), we performed PCAs and used the values of the first axes to represent ground cover and local microclimate. Prior to these analyses, we applied the Kaiser–Meyer–Olkin (KMO) measure of sampling adequacy to assess the appropriateness of the correlation matrices for PCAs. Each value was higher than 0.5, which is considered satisfactory for PCAs (Kaiser 1974; Table S4). To incorporate vegetation attributes, we ran two sets of models: first, we included plot-scale plant species richness (i.e., number of detected species, a measure of taxonomic diversity) and functional diversity (expressed by Rao’s Quadratic Entropy (RaoQ) values, Botta-Dukát, 2005; Fig. S1), while in the second one, we tested the effect of plant composition by performing a principal coordinate analysis (PCoA) on the plant species-abundance matrix and using the values of the first ordination axis. Finally, to describe ant communities, we included the plot-scale species richness and functional diversity (RaoQ values) in all the SEMs. We used Gaussian and Poisson error terms in the component models and included the location (wood-pasture) and sampling site IDs as nested random factors to account for potential spatial autocorrelation. To assess model goodness-of-fit and check for missing paths among unconnected variables, we used Shipley’s test of directed separation through Fisher’s C statistic, which is considered adequate with *p* > 0.05 (Shipley 2000). To optimize the SEMs, we used a manual backward selection procedure based on the corrected Akaike’s information criterion (AICc) and removed non-significant paths until reaching the final model with the lowest AICc value, while maintaining *p* > 0.05 (Lin et al. 2017).

To assess the adequacy of our sampling, we used sample-size and sample-completeness-based rarefaction and extrapolation curves (Hsieh et al. 2016). The asymptotic behavior of these curves (Figs. S2-S4) indicate that sampling effort was sufficient to support robust taxonomic and trait-based inferences. It should be noted, however, that certain ant species (e.g., rare species with cryptic lifestyles, or species with small colony sizes) might still be underrepresented in our study due to the limitations of baiting.

All analyses were performed in R (v. 4.2.2, R Core Team 2022). We used the *functcomp* function of the ‘FD’ package for calculating the CWMs (Laliberté et al. 2014), and the *gawdis* function of the ‘gawdis’ package for calculating the Gower distance matrices (de Bello et al. 2021). Pairwise PERMANOVAs were calculated using the *adonis2* function, and functional PCoAs were prepared with the *wcmdscale* function of the ‘vegan’ package (Oksanen et al. 2018). We used the *dudi.coa*, *dudi.pca*, *dudi.hillsmith*, *rlq* and *randtest* functions for the RLQ analyses and the *fourthcorner* function for the fourth-corner analyses of the ‘ade4’ package (Dray and Dufour 2007). The path models were built using the *psem* function of the ‘piecewiseSEM’ package (Lefcheck 2016). To construct component models for SEMs, we used the *lmer* and *glmer* functions of the ‘lme4’ package (Bates et al. 2014). The RaoQ values for SEMs were calculated with the *melodic* function provided by de Bello et al. (2016). We used the *KMO* function of the ‘psych’ package to test sampling adequacy for the ground cover and microclimate PCAs used in SEMs. We used the *iNEXT* function of the ‘iNEXT’ package (Hsieh et al. 2016) to create sample-size and sample-completeness-based rarefaction and extrapolation curves.

## Results

### Taxonomic and functional composition

Across the three wood-pastures and two seasons, we recorded a total of 187 plant species and 30 ant species–numbers consistent with previous studies in similar habitats (e.g., Tölgyesi et al. 2018; Tăușan et al. 2021). The taxonomic PERMANOVAs revealed substantial compositional differences among the different habitat types (grasslands, solitary trees, forest edges, and forests) for both plant and ant communities. For plants, each habitat type hosted compositionally unique communities during both seasons (spring and summer; *p* < 0.035; Table S5), with the only exception for forest edges and forests during summer (*p* = 0.094). For ants, grassland communities did not differ significantly from those of solitary trees (*p* > 0.198), while forest edge communities were similar to those found in forests (*p* > 0.278). Other combinations were significantly different from one another in both seasons (*p* < 0.002; Table S5).

**Fig. 2:**
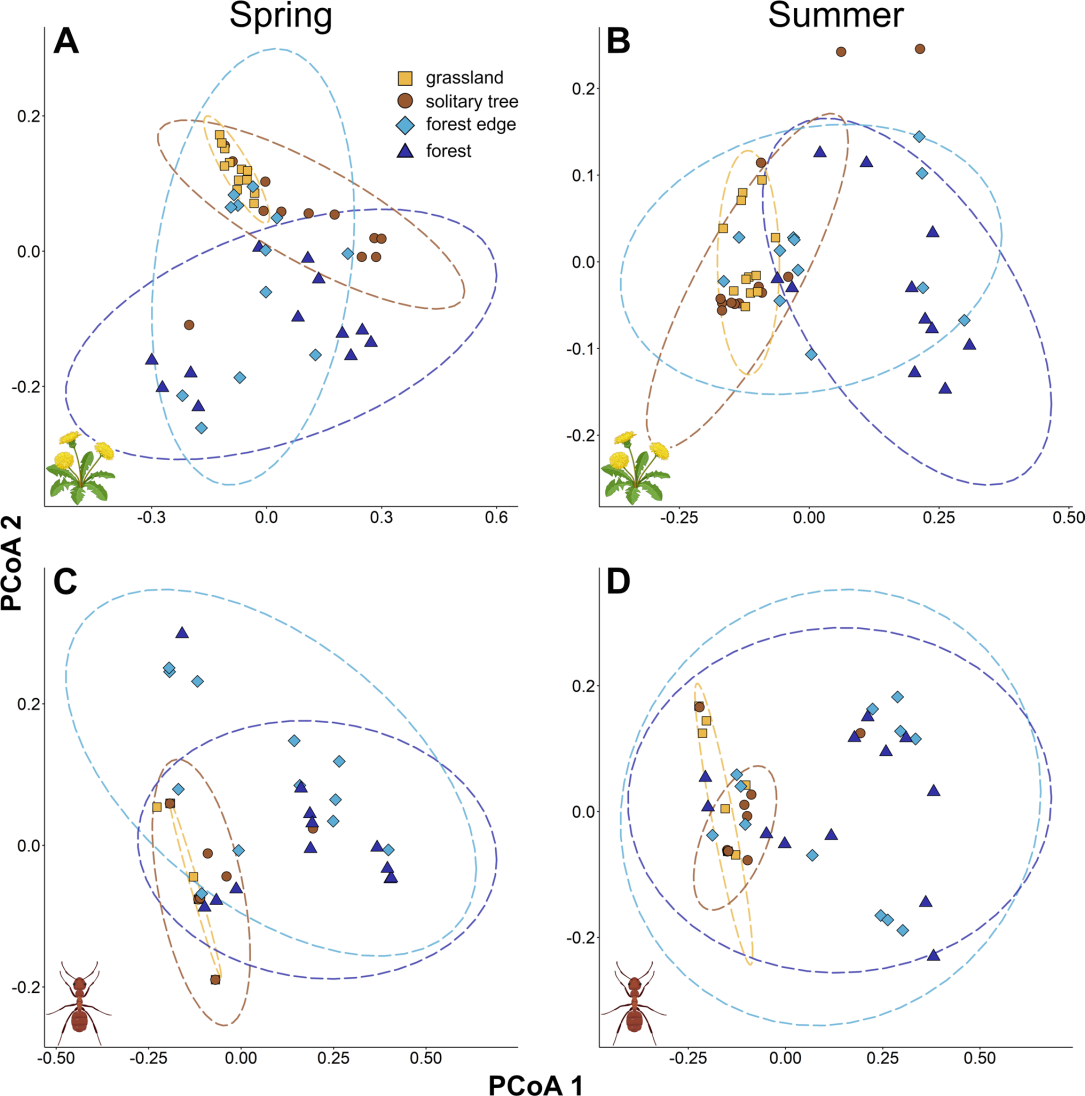
Functional composition of plant (**A**, **B**) and ant (**C**, **D**) communities at the four different habitat types of wood-pastures (grasslands, solitary trees, forest edges, and forests). Functional PCoA ordinations were performed for spring and summer separately. Ellipses were drawn at the 95% confidence intervals. Figures were illustrated using BioRender.com

The results of functional PERMANOVAs generally reflected similar patterns, indicating that the communities differed not only in species composition but also in functional characteristics (Fig. 2). For plants, we observed significant differences in functional trait composition among all the habitat types (*p* < 0.044; Table S6), except for forest edges and forests (*p* > 0.058). The grasslands differed significantly from solitary trees in spring (*p* = 0.006), however, this difference was no longer observed in summer (*p* = 0.390). Ant communities followed the same pattern as observed for taxonomic composition: grasslands were functionally similar to solitary trees (*p* > 0.204), and forest edges were similar to forests (*p* > 0.196), while all other combinations showed significant differences in both seasons (*p* < 0.009; Table S6). Based on the functional PCoAs, the samples from forest edges and forests were characterized by more diverse trait compositions than samples from grasslands and solitary trees for both plants and ants (Fig. 2). Samples in grasslands showed the most similar trait compositions among sampling units, as they occupied the smallest area in the ordination space and were mostly encompassed by solitary trees.

### Trait-environment associations

**Fig. 3:**
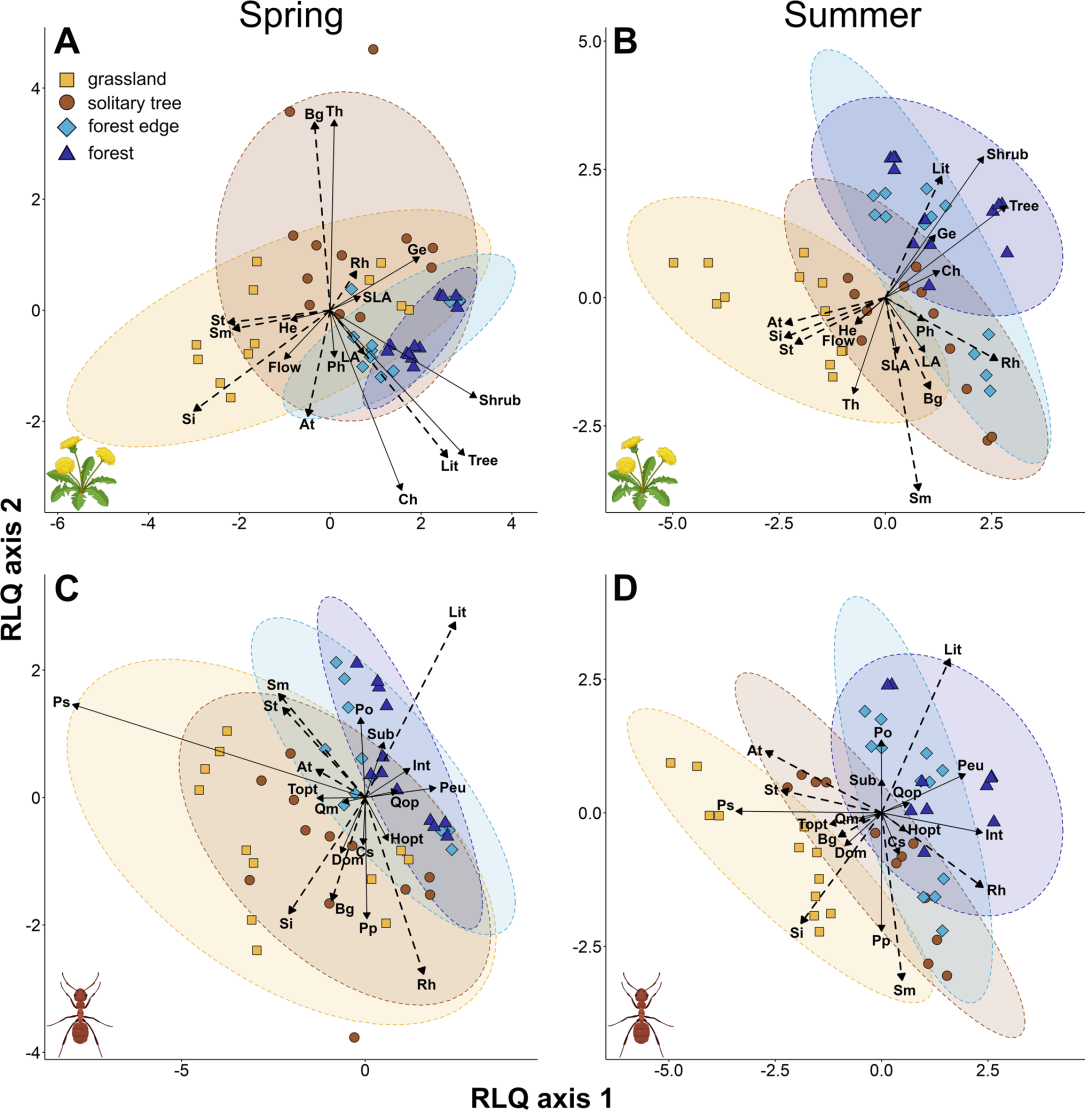
Ordination diagrams of the first two axes of the RLQ analyses displaying the environmental variables (dashed arrows) and functional traits (solid arrows) for plant (**A**, **B**) and ant (**C**, **D**) communities. Separate analyses were performed for spring and summer for both groups. Ellipses were drawn at the 95% confidence intervals. *Environmental variables*: *At* air temperature, *Bg* bare ground, *Lit* leaf litter, *Rh* relative air humidity, *Si* solar irradiation, *Sm* soil moisture, *St* soil temperature; *plant traits*: *Ch* chamaephyte, *Flow* flowering start, *Ge* geophyte, *He* hemicryptophyte, *LA* leaf area, *Ph* plant height, *SLA* specific leaf area, *Th* therophyte; *ant traits*: *Cs* cephalic size, *Dom* dominance–dominant, *Hopt* humidity optimum, *Int* dominance–intermediate, *Peu* ecological plasticity–eurytopic, *Po* ecological plasticity–oligotopic, *Pp* ecological plasticity–polytopic, *Ps* ecological plasticity–stenotopic, *Qm* queen number–monogynous, *Qop* queen number–oligogynous/polygynous, *Sub* dominance–subordinate, *Topt* temperature optimum. Figures were illustrated using BioRender.com

The RLQ ordination for plants showed that patterns of species composition were largely associated with local environmental conditions and variables related to ground cover. During spring, the RLQ explained a projected total inertia of 0.96. The first two axes accounted for 95.49% (68.23% and 27.26%, respectively) of the total variance. The eigenvalues of the first and second axes were 0.66 and 0.26, respectively. The global test revealed significant relationship between species distribution and environmental variables (model 2, *p* = 0.004) and marginally significant relationship between species composition and functional traits (model 4, *p* = 0.069). The negative (left) part of the first RLQ axis, mostly including grassland plots, identified hemicryptophytes and species with earlier flowering start (Fig. 3A). The occurrence of these species was positively correlated with higher levels of solar irradiation, soil temperature, and higher soil moisture levels (Table S7). The positive (right) part of the axis mainly included forest edge and forest plots characterized by higher LA values, and higher cover of shrubs, trees, and chamaephytes. These trait attributes were mainly associated with higher amounts of leaf litter (Table S7). The second RLQ axis was positively correlated with bare ground cover and identified solitary tree plots with higher cover of geophytes and therophytes. During summer, a projected total inertia of 1.77 was explained by the RLQ ordination. The first two axes explained 98.58% (93.03% and 5.55%, respectively) of the total variance. The eigenvalues of the first and second axes were 1.64 and 0.1, respectively. The multivariate test on the RLQ analysis indicated the global significance of the trait-environment relationships (model 2: *p* < 0.01; model 4: *p* < 0.01). The overall results in summer were similar to those observed in spring, with more environmental variables showing significant correlations with the individual RLQ axes (Fig. 3B, Table S7). Grassland plots, occupying the negative part of the first RLQ axis, harbored more therophytes, hemicryptophytes, and plants with an earlier start of flowering. These trait attributes were positively associated with higher irradiation levels, and higher air and soil temperatures. The positive part of the axis correlated with air humidity levels and the amount of leaf litter, and mainly included forest edge and forest plots characterized by higher cover of shrubs, trees, geophytes, and chamaephytes. The second RLQ axis was positively correlated with soil moisture levels and identified solitary tree and some forest edge plots with higher SLA and LA values.

The fourth-corner analyses also highlighted that associations between trait attributes and environmental conditions for plants were stronger during summer: we found only one significant bivariate association between functional traits and environmental variables in spring, and 18 of those were identified in summer (Table S8). In spring, hemicryptophytes were positively associated with solar irradiation. In summer, their occurrence was also positively influenced by air and soil temperature, while negatively influenced by relative air humidity and leaf litter. Plant height was negatively associated with solar irradiation, air and soil temperature, and positively associated with relative air humidity. LA was also negatively associated with air temperature, but positively associated with relative air humidity and soil moisture. Trees showed opposite trends compared to hemicryptophytes, as their occurrence was positively associated with relative air humidity and leaf litter, and negatively associated with solar irradiation and air temperature. Shrubs were positively associated with leaf litter, while the start of flowering was positively associated with soil temperature.

For ants, we found no significant associations between RLQ ordination axes and environmental variables (**Table S7**). Additionally, the fourth-corner analysis revealed no significant bivariate associations between ant functional traits and environmental variables across the two seasons, indicating that patterns of taxonomic and functional composition were not primarily influenced by local microclimate (**Table S8**). In spring, RLQ explained a projected total inertia of 1.68. The first two axes accounted for 93.57% (71.15% and 22.42%, respectively) of the total variance. The eigenvalues of the first and second axes were 1.2 and 0.38, respectively. In summer, a projected total inertia of 1.42 was explained by the RLQ ordination. The first two axes accounted for 93.81% (80.57% and 13.24, respectively) of the total variance. The eigenvalues of the first and second axes were 1.15 and 0.19, respectively. In both seasons, the global tests revealed significant relationships only between ant species distribution and environmental variables (model 2: *p* < 0.001). There were no significant relationships between species composition and functional traits (model 4: spring: *p* = 0.661, summer: *p* = 0.494). Reflecting the results of the taxonomic and functional PERMANOVAs, the first RLQ axis separated the habitat types into two groups in both seasons: grasslands were more similar to solitary trees, while forest edges were more similar to forests (Fig. 3C, D). Grasslands and solitary trees hosted more polytopic, stenotypic, monogynous, and dominant ants, with higher temperature optima. Although causality cannot be implied, these trait attributes were generally associated with increased temperature and solar irradiation levels. In contrast, forest edges and forests had more subordinate, intermediate, oligogynous, polytopic and eurytopic ants, and species with higher cephalic sizes. These trait attributes were generally associated with lower temperatures, and higher air humidity levels and leaf litter cover.

### Direct and indirect factors influencing ant diversity metrics

**Fig. 4:**
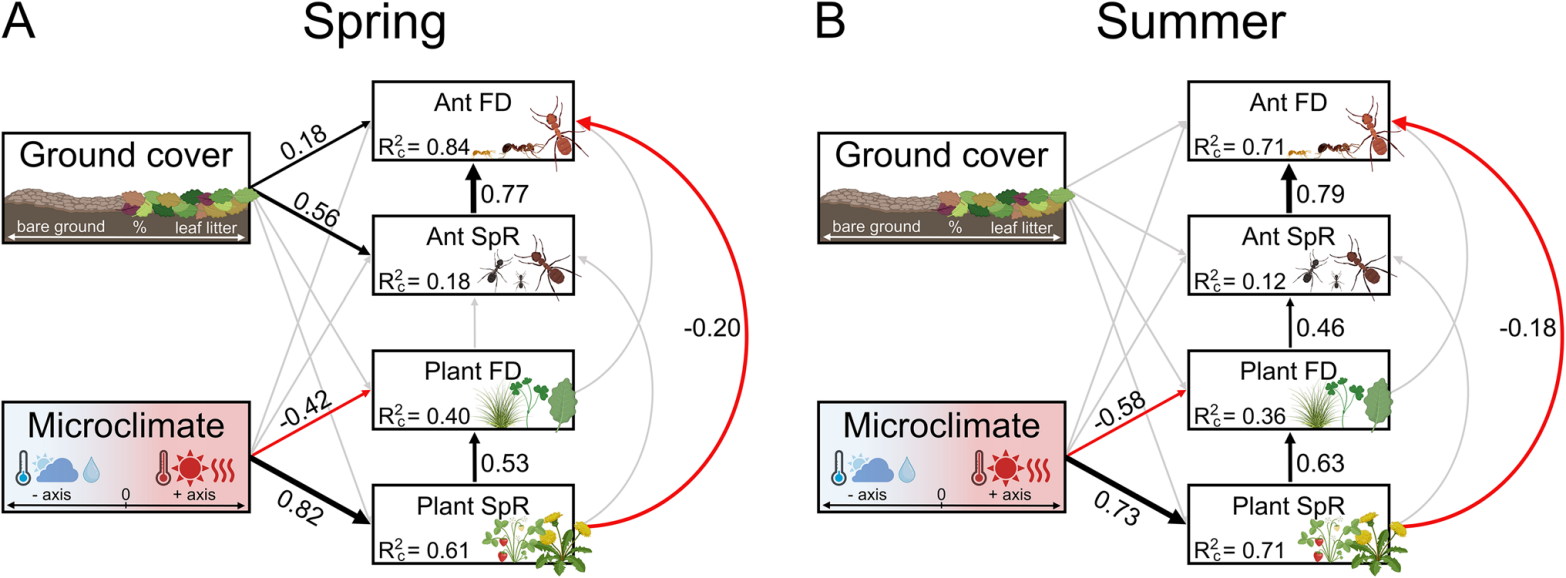
Structural equation models (SEMs) exploring the effects of local microclimate and ground cover (soil surface complexity) on plant and ant diversity metrics (species richness and functional diversity) during **A** spring, and **B** summer. Black arrows represent positive, while red arrows represent negative significant unidirectional relationships among variables. Non-significant paths (*p* > 0.05) are represented with gray arrows. Arrow thickness is proportional to the standardized regression coefficient, denoted next to the arrows. Conditional R^2^ values for component models are given with the response variables. *FD* functional diversity (expressed by Rao’s quadratic entropy), *SpR* species richness. Path diagrams were created using BioRender.com

The path analyses revealed that ant diversity metrics (taxonomic and functional diversity) were strongly influenced by local-scale habitat characteristics, vegetation-related variables, and microclimate through direct and indirect pathways (Fig. 4). The strength and structure of relationships showed differences between seasons, suggesting that the communities were primarily shaped by different processes during spring and summer. Based on R^2^ values derived from the variance of both fixed and random effects (conditional R^2^), the models including plant species richness and functional diversity explained a slightly larger proportion of variance than those including plant species composition (Table S9). During spring, local ground cover (i.e., high leaf litter cover and low bare ground cover) had a positive direct effect on ant species richness and functional diversity (Fig. 4A). This effect disappeared during summer, when ant diversity metrics were primarily shaped by vegetation characteristics: plant species richness negatively affected ant functional diversity (this was also observed in spring), while plant functional diversity had a direct positive effect on ant species richness (Fig. 4B). Local microclimate indirectly influenced ant diversity metrics in both seasons through its strong positive effect on plant species richness and negative effect on plant functional diversity (i.e., hot and sunny conditions with low air humidity levels promoted plant species richness but decreased functional diversity). Finally, species richness positively influenced functional diversity for both plants and ants during each season, although this relationship was consistently stronger for ants.

## Discussion

To our knowledge, our study is the first to provide a comprehensive understanding of the direct and indirect processes shaping the distribution of taxonomic and functional composition and diversity across different trophic levels (plants and ants) in complex landscapes. Our results show that the four different habitat types of wood-pastures (grasslands, solitary trees, forest edges, and forests) have the capacity to sustain distinct plant and ant communities with unique taxonomic and functional compositions, thereby boosting landscape-level biodiversity. However, while both plant and ant communities mapped the increased environmental heterogeneity provided by the different habitat types, the primary mechanisms driving their community organization differed. For plants, compositional patterns and trait distributions were largely influenced by local environmental conditions, as indicated by the multiple significant relationships between environmental variables and trait attributes. The trait-environment associations were generally stronger during summer, when the environmental contrast among the habitat types was larger. For ants, on the other hand, we did not detect any significant direct trait-environment associations, and local microclimate alone failed to explain compositional patterns and trait distributions. Instead, ant diversity metrics (species richness and functional diversity) were indirectly affected by microclimate through its strong effect on vegetation.

The observed differences in the taxonomic composition of plant and ant communities align with previous studies from similar habitats (e.g., Tölgyesi et al. 2018; Gaytán et al. 2021; Tăușan et al. 2021). Importantly, these compositional differences were reflected at the functional level, highlighting the potential of complex landscapes to promote not only the taxonomic but also the functional diversity at the landscape scale. The primary drivers governing the distribution of taxonomic and functional attributes, however, differed for ants and plants. Our results suggest that vegetation compositional patterns and functional trait distributions were largely and directly associated with local environmental conditions. For instance, we found that hemicryptophytes were associated with sunny and warm conditions (found mainly on grasslands) during both seasons, as these species are adapted to areas with higher light availability, and are resilient to grazing and frost due to their renewal buds located at the soil surface (Midolo et al. 2024). Moreover, species under these conditions were characterized by smaller leaf areas and specific leaf areas (i.e., thicker leaves), indicating the predominance of conservative resource-use strategies and defense mechanisms against herbivores (Díaz et al. 2004; Maracahipes et al. 2018). In contrast, the shaded, cooler, and humid sites of forest edges and forests were characterized by high covers of shrubs and tree saplings. Species under these conditions tended to be taller, enabling them to capture and utilize resources such as light more efficiently (Lavorel and Garnier 2002; Díaz et al. 2004), and to penetrate the thick leaf litter layer (Loydi et al. 2014). Meanwhile, solitary trees hosted plant species with larger leaves and higher specific leaf area, which indicate higher resource capture ability and utilization capacity (Díaz et al. 2004; Pellegrini 2016). The dominance of such traits is likely linked to the combined effect of microclimatic context and resource availability at these habitat types. The canopy of solitary trees creates cooler and moister conditions and unique insolation regimes for the understory vegetation (Lőrincz et al. 2024a), mitigating water stress, which is generally responsible for reduced leaf area and specific leaf area in plants (Wright et al. 2005). Additionally, nutrient concentrations are often elevated under trees due to the combined effects of litter decomposition, root turnover, and most importantly, manure deposition by grazing livestock seeking shelter beneath the canopy (Manning et al. 2006; Tölgyesi et al. 2023). The latter is also responsible for the high levels of bare ground beneath trees, as concentrated animal presence leads to soil trampling and increases grazing pressure (Tölgyesi et al. 2018).

Plant trait-environment associations were generally stronger during summer, with 18 significant associations observed, compared to only one in spring. This pattern likely reflects the seasonal variability of key environmental parameters influencing plant distributions. While some resources, such as soil nutrients vary mostly in space, and are mainly affected by topography (e.g., habitat type), others, including light availability and soil moisture content, vary both spatially and temporally due to seasonal changes in overstory canopy cover (Seibert et al. 2007; Barbier et al. 2008). During spring, before canopy closure, light availability and soil moisture content is high at each habitat type (even during rain-free periods). Under these conditions, the understory herb layer is mainly shaped by the abiotic and edaphic characteristics of each habitat type, as well as a mixture of facilitative and competitive interactions between the under- and overstory vegetation (Tölgyesi et al. 2023). In summer, however, the fully developed tree canopy severely limits light availability for understory vegetation and reduces local soil moisture through canopy interception and transpiration, particularly in areas with high canopy covers (e.g., forest edges and forests) (Lőrincz et al. 2024a). The increased environmental contrast and resource limitation likely exerts a strong filter on vegetation and thus accounts for the stronger trait-environment associations observed during summer.

Contrary to plants, for ants, we did not find any direct relationships between local microclimate and diversity metrics. Instead, our results indicate that microclimate indirectly affected the patterns of the taxonomic and functional composition of ants by affecting vegetation characteristics. Although this may initially seem surprising given the well-documented role of microclimate in shaping ant activity patterns and coexistence mechanisms (Cerdá et al. 1997; Philpott et al. 2010), multiple studies have reported similar findings for a wide range of taxa, including ants. Vegetation attributes have been shown to predict taxonomic composition and trait variation more efficiently than environmental or structural variables for herbivores, such as grasshoppers (van der Plas et al. 2012), weevils, planthoppers, and spittlebugs (Schaffers et al. 2008). The same pattern was observed at higher trophic levels among omnivorous and predatory taxa (spiders, carabids: Schaffers et al. 2008; ants: Frenette-Dussault et al. 2013), indicating that strong bottom-up effects can occur without obligate or highly specialized relationships with vegetation. In fact, vegetation attributes, such as species richness and functional diversity or composition encapsulate and synthesize a wide range of causal factors relevant for arthropods across different trophic levels (Schaffers et al. 2008). Plant taxonomic and functional composition itself is determined by, and therefore, summarizes local environmental conditions, as well as disturbance and/or management regimes (Klimek et al. 2007). The established vegetation, in turn, determines the vegetation structure, which affects the local microclimate and influences the quality and quantity of resources available to arthropods for feeding, nesting, and oviposition (van Klink et al. 2015). The used plant diversity metrics (species richness and functional diversity), therefore, integrate several components critical for arthropods, which might explain their central position in explaining ant diversity metrics (Frenette-Dussault et al. 2013).

Our path models revealed that warm and sunny conditions promoted plant species richness, which in turn negatively influenced ant functional diversity during both seasons. Accordingly, we detected the lowest ant functional diversity and highest plant species richness in grasslands, the most open habitat type. It is important to note, however, that our species-rich grasslands exhibited low functional diversity and high functional homogeneity (lowest RaoQ values and smallest occupied area in the functional ordination space), reflecting the similar adaptations of plant species to the harsh environmental conditions and grazing (aligning with the stress-dominance hypothesis; Weiher and Keddy 1995). The combination of these factors might impact ants in contrasting ways. On one hand, the warm microclimate benefits brood development and colony growth (Hölldobler and Wilson 1990), which is facilitated by the abundant food sources, such as plant seeds and honeydew produced by subterranean and epigeous hemipterans. The homogeneous habitat structure, however, severely limits nesting site options and diversity of food resources, leaving little room for niche differentiation among species (Lőrincz et al. 2024a). This combination likely selects for a few functionally similar ant species with good competitive abilities, explaining the observed low ant functional diversity, low species numbers, and high proportion of dominant species in our study.

Besides species richness, the functional diversity of vegetation also affected ant diversity metrics through direct and indirect pathways. Specifically, plant functional diversity positively influenced the species richness of ants, which in turn enhanced functional diversity within ant communities. This connection, however, was only observed during summer. Plant functional traits, such as growth form, height, leaf area, or start of flowering play an important role in determining microhabitat conditions by influencing vegetation structure, litter formation and retention, and local microclimate (Chillo et al. 2017). Variability in such traits increases the diversity in nesting sites, foraging substrates, food resources, and microclimatic conditions for ants (van Klink et al. 2015), and thereby facilitating species coexistence by promoting niche differentiation (Cerdá et al. 2013). Indeed, the proportion of oligotopic and subordinate species increased in habitat types with high functional diversity and structural complexity of plants, such as forest edges and forests (occupying the largest area in the functional ordination space), where representatives of these groups have been shown to exhibit narrow realized niche breadths and small niche overlaps with dominant species (Lőrincz et al. 2024a, b). In spring, instead of plant functional attributes, soil surface complexity seemed to influence ant diversity metrics. Given that the used vegetation attributes did not change substantially across seasons, ground cover likely played a more fundamental role in shaping ant communities under springtime conditions, partially overriding the effects conveyed by vegetation. This might be explained by multiple non-mutually exclusive mechanisms. In spring, cold temperatures severely restrict ant activity, resulting in significantly lower species numbers compared to summer across all habitat types (Lőrincz et al. 2024b). Under these conditions, a developed litter layer might foster ant activity by buffering temperature fluctuations and protecting ants against wind (Sayer 2006). Moreover, as spatial patterns of litter accumulation align with those of plant functional diversity, litter cover might function as a surrogate for habitat structure during spring, when vegetation is less developed.

Finally, we found stronger relationships between species richness and functional diversity for ants compared to plants in both seasons. As the strength of this relationship is dependent upon community functional redundancy (Cadotte et al. 2011), this result might allow for further inferences regarding the organization of the studied plant and ant communities. For vegetation, the lower correlation may indicate higher functional redundancy (i.e., lower functional trait dissimilarity) due to trait convergence caused by the combined effects of grazing and environmental constraints (Carmona et al. 2012; Chillo et al. 2017). In contrast, the strong correlation detected for ants implies higher niche differentiation and niche complementarity, with each species harboring unique trait values and attributes, and therefore, expanding community-level trait space (i.e., lower functional redundancy) (Chillo et al. 2017). This may further emphasize that the primary structuring force seems to differ for ant and plant communities (i.e., interspecific competition versus environmental filtering), which likely accounts for the incongruent patterns in the spatial distribution and peaks of their species richness and functional diversity.

## Conclusions

Using wood-pastures as a model system, and plants and ants to represent different trophic levels, our study demonstrated that the high environmental heterogeneity of these landscapes has the potential to boost landscape-level taxonomic and functional diversity at different trophic levels. While both plant and ant communities reflected the heterogeneity of the system, the main mechanisms driving their functional trait distributions and diversity metrics were fundamentally different. Plant taxonomic and functional composition was primarily shaped by local environmental conditions, whereas for ants, direct associations between environmental conditions and trait values or attributes were not detected. Instead, their diversity metrics and compositional patterns were mainly influenced by vegetation and habitat characteristics. As a result, the peaks of taxonomic and functional diversity of plants and ants did not align in space across different habitat types, as certain vegetation diversity metrics contrastingly influenced the diversity metrics of ants, and possibly other organisms along the food-web. This spatial mismatch reinforces the “ecosystem complex” approach of heterogeneous landscapes, emphasizing that conservation initiatives should focus on the system as a whole, rather than individual habitat types, to maximize biodiversity conservation.

## Supplementary Information

Below is the link to the electronic supplementary material.

Supplementary file1 (DOCX 1003 KB)

## Data Availability

The datasets used and/or analyzed during the current study are deposited in Zenodo: 10.5281/zenodo.17153658.
