# Supplementary material for: Contrasting trait-based assembly mechanisms on different trophic levels: ants and plants on wood-pastures
Source: Oecologia. 2025 Sep 30;207(10):166. doi: 10.1007/s00442-025-05802-4 (PMC12479661; doi:10.1007/s00442-025-05802-4)
Supplement: Supplementary file 1 — Supplementary file1 (DOCX 1003 KB) [file 442_2025_5802_MOESM1_ESM.docx]

**Supplementary material for:**

**Contrasting trait-based assembly mechanisms on different trophic levels: ants and plants on wood-pastures**

Ádám Lőrincz, Kata Frei, Alida Anna Hábenczyus, Bonita Ratkai, Gábor Lőrinczi, András Kelemen, Csaba Tölgyesi, Zoltán Bátori, István Elek Maák

**Table of Contents:**

| **Figure S1.** Functional diversity of plant and ant communities at the four different habitat types of wood-pastures | Page 2 |
| --- | --- |
| **Figure S2.** Sample-size and sample-completeness-based rarefaction and extrapolation sampling curves for plants and ants – data pooled across habitat types | Page 3 |
| **Figure S3.** Sample-size and sample-completeness-based rarefaction and extrapolation sampling curves for plants – data shown for the individual habitat types separately | Page 4 |
| **Figure S4.** Sample-size and sample-completeness-based rarefaction and extrapolation sampling curves for ants – data shown for the individual habitat types separately | Page 5 |
| **Table S1.** General characteristics of the three wood-pastures selected for our study | Page 6 |
| **Table S2.** Details of the plant and ant functional traits used for the study | Page 7 |
| **Table S3.** Pearson correlation coefficients among microclimate and ground cover values | Page 8 |
| **Table S4.** Kaiser-Meyer-Olkin (KMO) measure of sampling adequacy for PCAs | Page 9 |
| **Table S5.** Composition of plant and ant communities – pairwise PERMANOVA | Page 10 |
| **Table S6.** Functional composition of plant and ant communities – pairwise PERMANOVA | Page 10 |
| **Table S7.** Correlation coefficients of environmental variables with the first and second RLQ axes for plants and ants | Page 11 |
| **Table S8.** Fourth-corner analysis – bivariate associations between plant and ant functional traits and environmental variables | Pages 12-14 |
| **Table S9.** Results of SEMs with different plant metrics | Page 15 |

**Figure S1.** Functional diversity (expressed by Rao’s Quadratic Entropy (RaoQ)) of plant and ant communities at the four different habitat types of wood-pastures (i.e., grasslands, solitary trees, forest edges, and forests). Different lowercase letters indicate significant differences between habitat types (*p* < 0.05). Values were compared by linear mixed-effects models, with habitat type included as a fixed factor and location (wood-pasture) as a random factor. The *p*-values were adjusted for multiple comparisons with the Tukey HSD method. We built separate models for both plants and ants at each season (spring and summer).

**
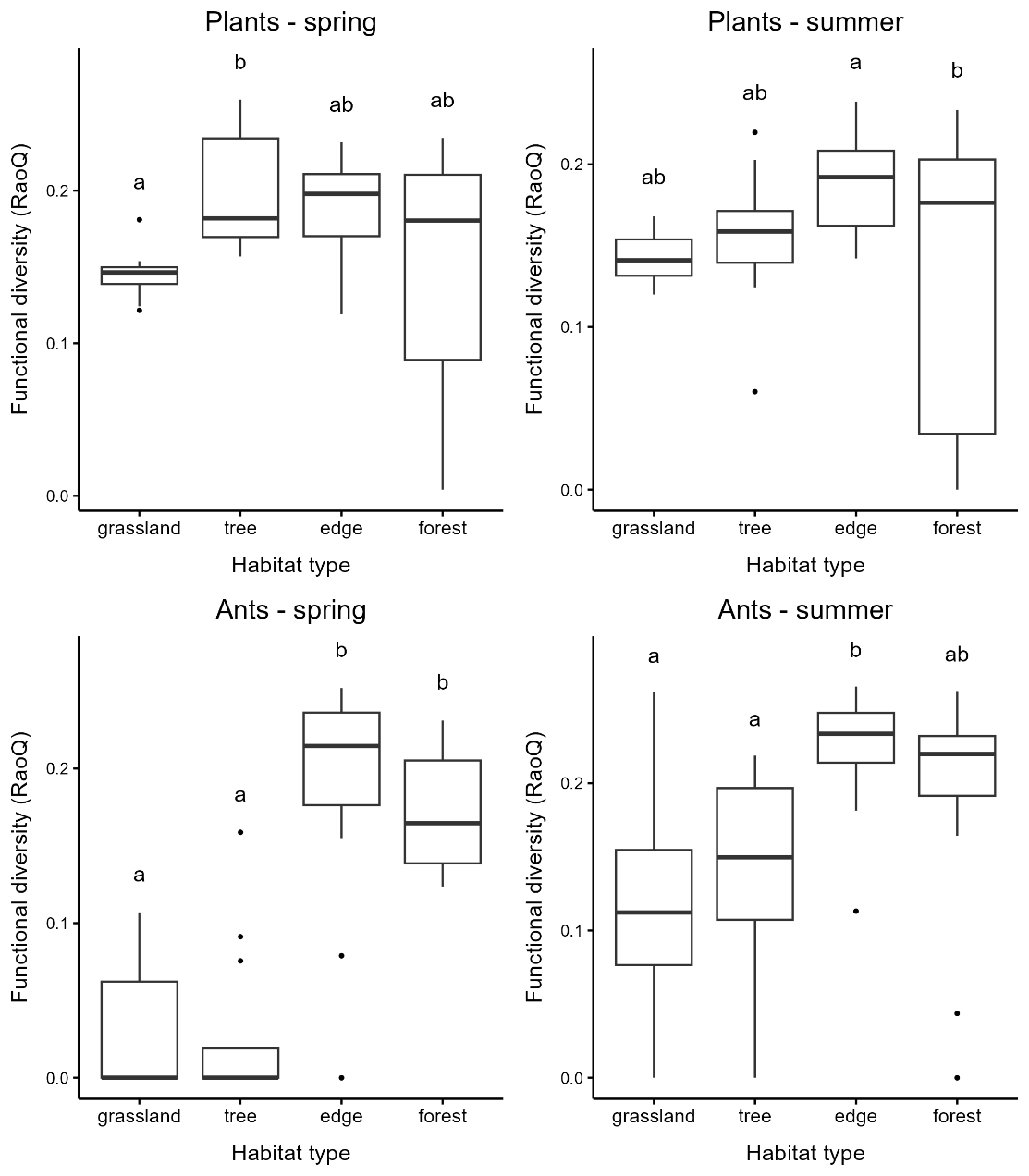
**

**Figure S2.** Sample-size and sample-completeness-based rarefaction (solid lines) and extrapolation (dashed lines) sampling curves with 95% confidence intervals for plants (A-B) and ants (C-D). Samples from the four different habitat types of wood-pastures (grasslands, solitary trees, forest edges, and forests) were pooled for both groups. Orange lines represent data collected in spring, while blue lines represent data collected in summer.

**
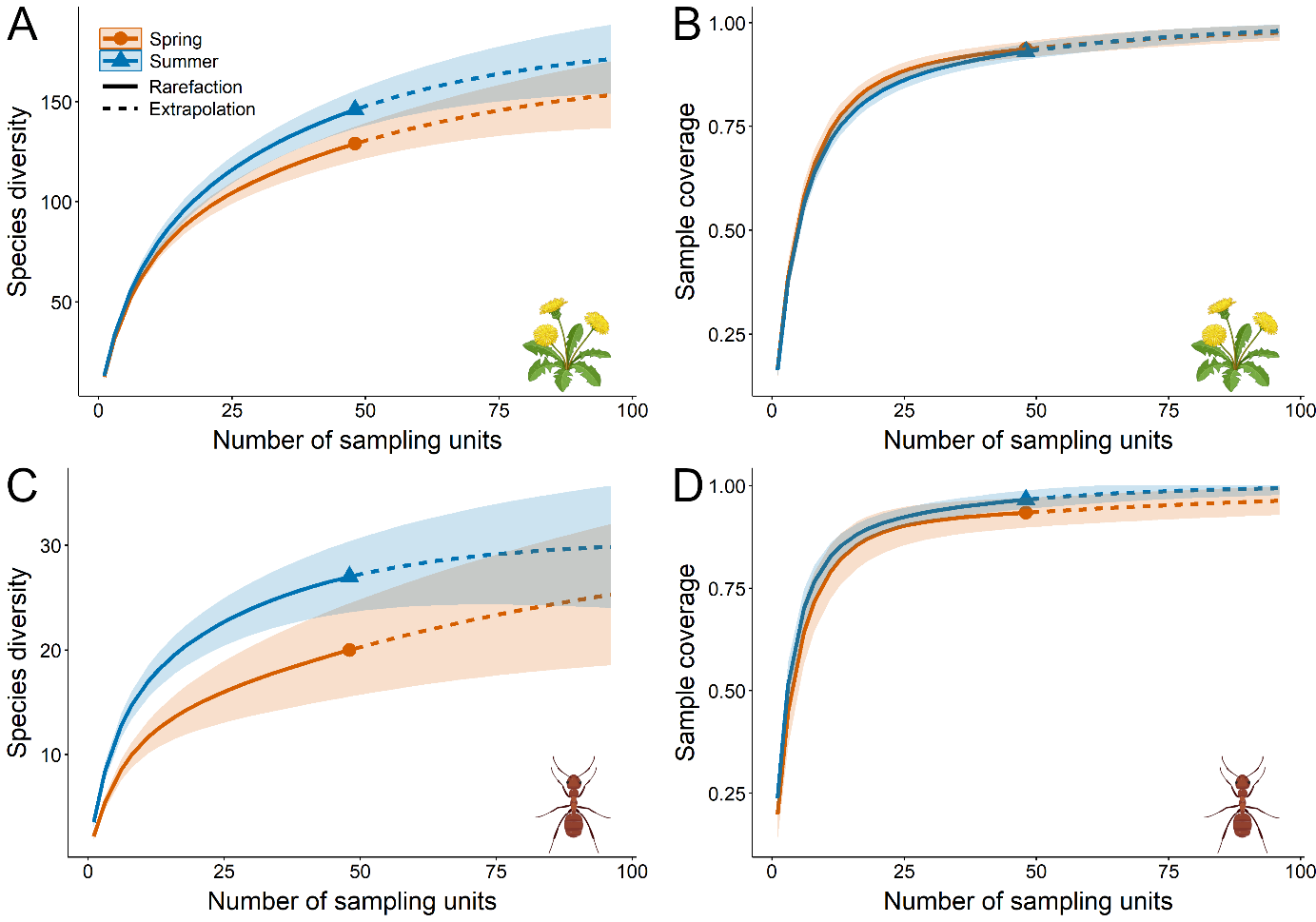
**

**Figure S3.** Sample-size and sample-completeness-based rarefaction (solid lines) and extrapolation (dashed lines) sampling curves with 95% confidence intervals for plants in spring (A-B) and summer (C-D). Samples from the four different habitat types of wood-pastures (grasslands, solitary trees, forest edges, and forests) were analyzed separately.

**
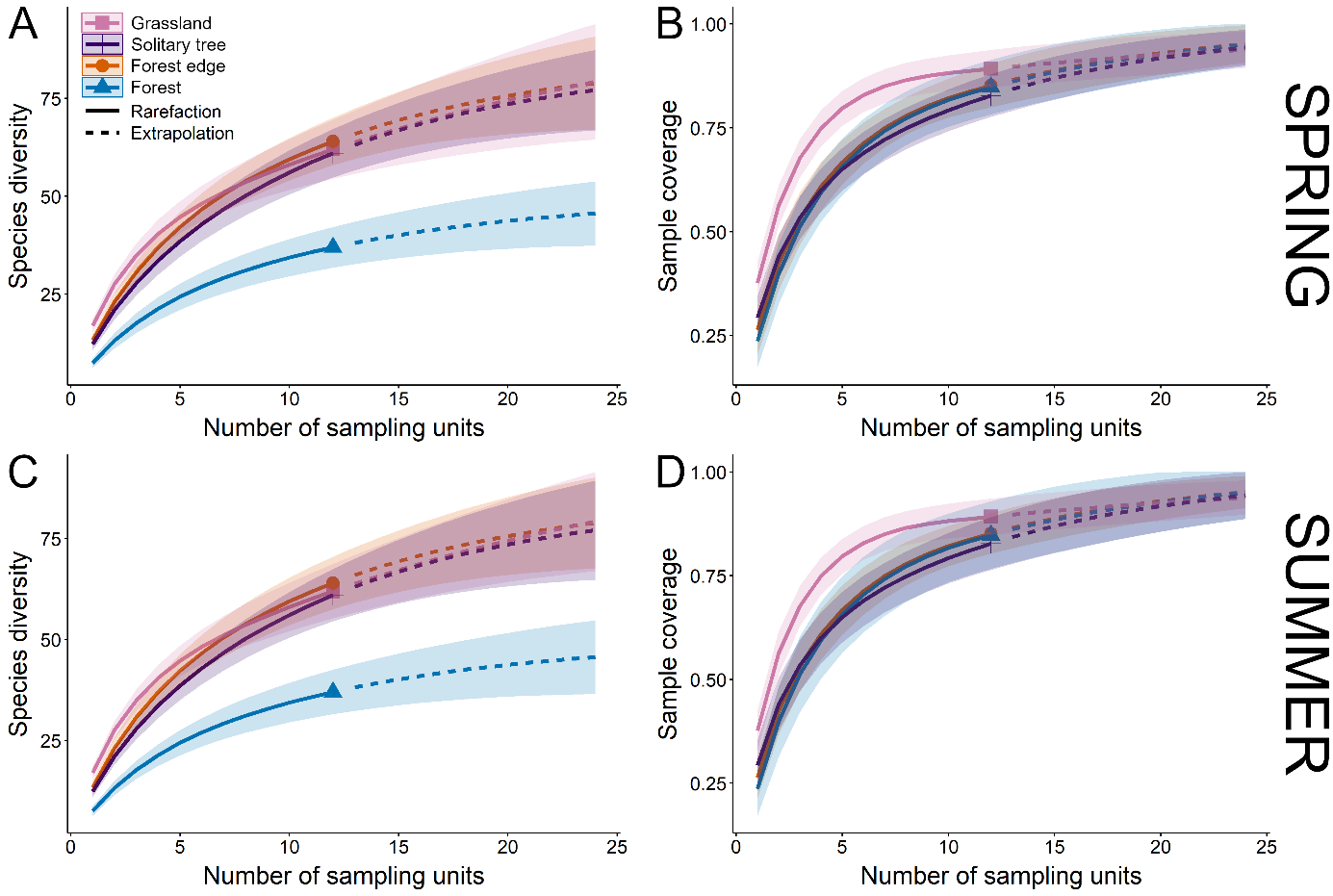
**

**Figure S4.** Sample-size and sample-completeness-based rarefaction (solid lines) and extrapolation (dashed lines) sampling curves with 95% confidence intervals for ants in spring (A-B) and summer (C-D). Samples from the four different habitat types of wood-pastures (grasslands, solitary trees, forest edges, and forests) were analyzed separately.

**
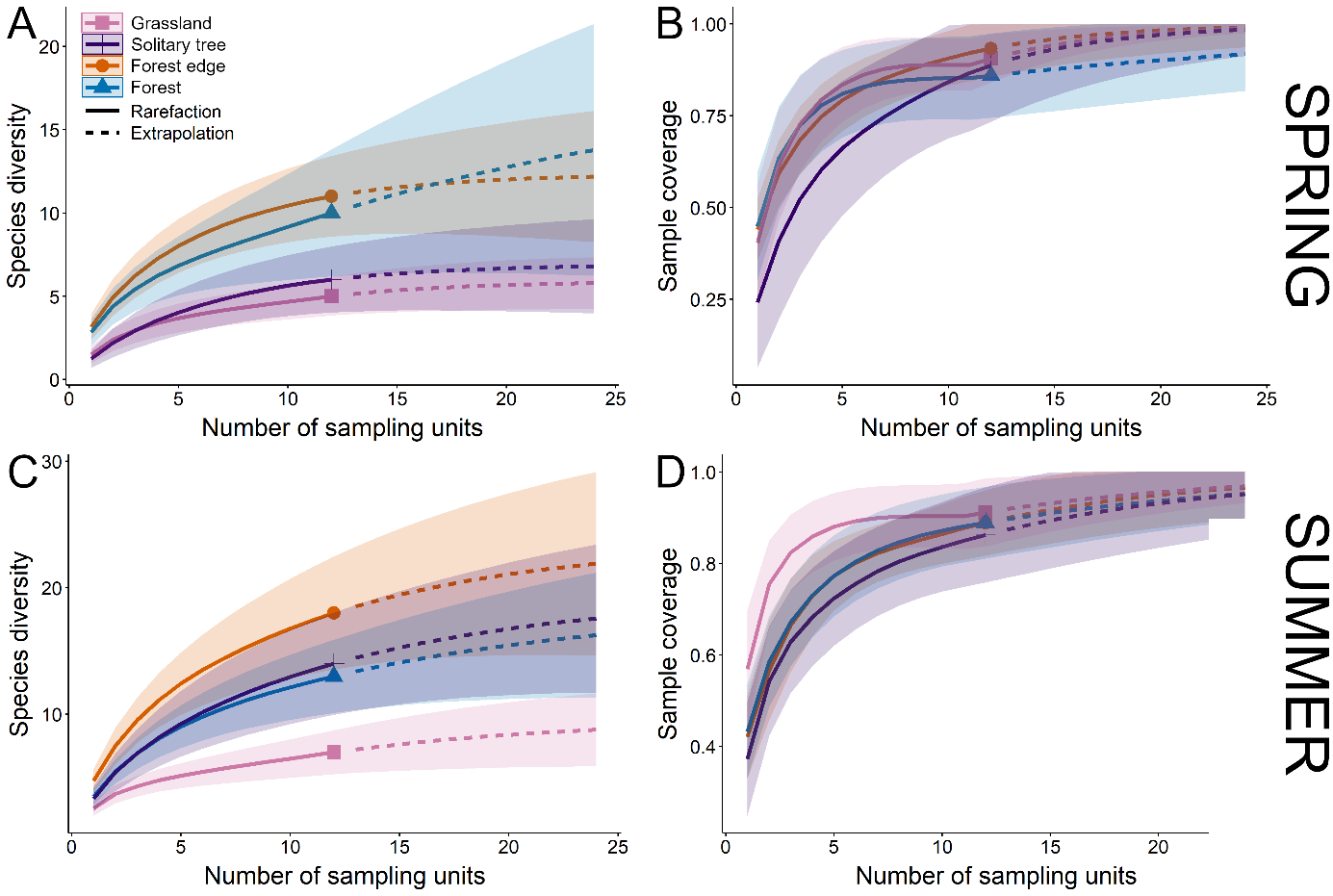
**

**Table S1.** Location and environmental characteristics of the three wood-pastures selected for our study.

| **Location** | **GPS coordinates** | **Area (Ha)** | **Elevation  (m a.s.l.)** | **Mean annual precipitation (mm)** | **Mean annual temperature (°C)** |
| --- | --- | --- | --- | --- | --- |
| Erdőbénye (Hungary) | N 48.276, E 21.319 | 215 | 215–245 | 600 | 9.5 |
| Deușu (Romania) | N 46.916, E 23.505 | 250 | 420–490 | 600 | 8.3 |
| Rupea (Romania) | N 46.019, E 25.223 | 393 | 500–580 | 650 | 8.2 |

**Table S2.** Details of the plant and ant functional traits used for the study.

| **PLANTS** | | | |
| --- | --- | --- | --- |
| **Trait name** | **Description** | **Data type** | **Source** |
| Plant height | Mean height of the aboveground shoot of adult plants | Numeric (cm) | Sonkoly et al. (2023) |
| Specific leaf area (SLA) | The ratio of leaf area to leaf dry weight | Numeric (mm^2^/mg) | Sonkoly et al. (2023) |
| Leaf area (LA) | One-sided area of a fresh leaf | Numeric (mm^2^) | Sonkoly et al. (2023) |
| Start of flowering | The month in which the species starts flowering | Numeric (month) | Sonkoly et al. (2023) |
| Life form | Raunkiær’s life form of the species, based on the perennating organs (seeds or buds) and the vertical position of buds | Nominal with six levels: chamaephytes, geophytes, shrubs, trees, hemicryptophytes, therophytes | Sonkoly et al. (2023) |
|  |  |  |  |
| **ANTS** | | | |
| **Trait name** | **Description** | **Data type** | **Source** |
| Cephalic size | Proxy for body size | Numeric (μm) | Seifert (2018) |
| Queen number | Number of egg-laying queens in a colony | Nominal with two levels: monogynous,  oligo-/polygynous | Czechowski et al. (2012), Seifert (2018) |
| Dominance | The capability of a species to exclude others from resources | Nominal with three levels: dominant, intermediate, subordinate | Based on direct measurements and observations (Lőrincz et al. 2024a,b) |
| Temperature optimum | Value of air temperature at which maximal foraging activity is observed | Numeric (°C) | Based on direct measurements (Lőrincz et al. 2024b) |
| Humidity optimum | Value of relative air humidity at which maximal foraging activity is observed | Numeric (%) | Based on direct measurements (Lőrincz et al. 2024b) |
| Plasticity | The extent of variation in ecological, environmental, and habitat conditions that a species can tolerate | Nominal with four levels: eurytopic, polytopic, oligotopic, stenotopic | Czechowski et al. (2012) |
|  |  |  |  |

**Note:** ***monogynous****: one egg-laying queen per colony;* ***oligo-/polygynous****: more than one egg-laying queen per colony;* ***dominant****: high behavioral and ecological dominance;* ***intermediate****: high behavioral, but less pronounced ecological dominance;* ***subordinate****: low behavioral and ecological dominance;* ***eurytopic****: species which show no clear preference for any type of habitat (open or closed) or ecological factor;* ***polytopic****: species which occur in many different habitats within their definite category (e.g., various types of forests);* ***oligotopic****: species occurring in habitats of a few similar types (e.g., various coniferous forests), or demand a specific physical factor, irrespective of the general type of vegetation;* ***stenotopic****: species closely associated with a habitat of one type (e.g., xerothermic grasslands).* ***Reference****: Sonkoly J, Tóth E, Balogh N, Balogh L, Bartha D, Csendesné Bata K et al (2023) PADAPT 1.0 – the Pannonian dataset of plant traits. Scientific Data 10:742. DOI: 10.1038/s41597-023-02619-9*

**Table S3.** Pearson correlation coefficients (r) among microclimate variables (range and mean) and among ground cover variables. Upper and lower triangles represent values for spring and summer, respectively. Abbreviations: AirHum-relative air humidity; AirTemp-air temperature; Irrad-irradiation; SoilTemp-soil temperature.

|  | **Microclimate variables** | | | | | | | | |
| --- | --- | --- | --- | --- | --- | --- | --- | --- | --- |
|  | **IrradRange** | **AirTempRange** | **AirHumRange** | **SoilTempRange** | **IrradMean** | **AirTempMean** | **AirHumMean** | **SoilTempMean** | **SoilMoisture** |
| **IrradRange** |  | 0.266 | -0.104 | 0.477 | 0.831 | 0.311 | -0.077 | 0.39 | 0.55 |
| **AirTempRange** | 0.743 |  | 0.784 | 0.294 | 0.347 | 0.028 | 0.494 | -0.271 | -0.334 |
| **AirHumRange** | 0.432 | 0.777 |  | 0.194 | 0.101 | 0.102 | 0.396 | -0.269 | -0.473 |
| **SoilTempRange** | 0.766 | 0.531 | 0.074 |  | 0.84 | 0.572 | -0.09 | 0.604 | 0.157 |
| **IrradMean** | 0.9 | 0.745 | 0.401 | 0.83 |  | 0.551 | -0.108 | 0.579 | 0.374 |
| **AirTempMean** | 0.428 | 0.315 | -0.189 | 0.721 | 0.583 |  | -0.757 | 0.777 | 0.49 |
| **AirHumMean** | -0.442 | -0.352 | 0.133 | -0.672 | -0.509 | -0.888 |  | -0.739 | -0.666 |
| **SoilTempMean** | 0.554 | 0.29 | -0.246 | 0.868 | 0.669 | 0.908 | -0.796 |  | 0.634 |
| **SoilMoisture** | -0.033 | -0.016 | 0.183 | -0.203 | -0.014 | -0.453 | 0.566 | -0.303 |  |

|  | **Ground cover variables** | |
| --- | --- | --- |
|  | **BareGroundCover** | **LeafLitterCover** |
| **BareGroundCover** |  | -0.506 |
| **LeafLitterCover** | -0.502 |  |
|  |  |  |

**Table S4.** Kaiser-Meyer-Olkin (KMO) measure of sampling adequacy (MSA) for the full model and for each variable separately for spring and summer. Abbreviations: AirHum-relative air humidity; AirTemp-air temperature; Irrad-irradiation; SoilTemp-soil temperature.

|  |  | **Spring** | | **Summer** | |
| --- | --- | --- | --- | --- | --- |
|  |  | **MSA for each variable** | **Overall MSA** | **MSA for each variable** | **Overall MSA** |
| **Ground cover variables** | BareGroundCover | 0.5 | 0.5 | 0.5 | 0.5 |
|  | LeafLitterCover | 0.5 |  | 0.5 |  |
| **Microclimate variables** | IrradRange | 0.54 | 0.64 | 0.79 | 0.75 |
|  | AirTempRange | 0.73 |  | 0.77 |  |
|  | AirHumRange | 0.51 |  | 0.58 |  |
|  | SoilTempRange | 0.62 |  | 0.80 |  |
|  | IrradMean | 0.64 |  | 0.80 |  |
|  | AirTempMean | 0.58 |  | 0.68 |  |
|  | AirHumMean | 0.54 |  | 0.82 |  |
|  | SoilTempMean | 0.78 |  | 0.72 |  |
|  | SoilMoisture | 0.84 |  | 0.65 |  |

**Table S5.** Pairwise comparisons of plant and ant community compositions in the four habitat types of wood-pastures with permutational multivariate analysis of variance (PERMANOVA). The *p*-values were adjusted with the FDR method. Significant differences (*p* < 0.05) are marked with boldface.

|  | PLANTS | | | | ANTS | | | |
| --- | --- | --- | --- | --- | --- | --- | --- | --- |
|  | Spring | | Summer | | Spring | | Summer | |
|  | F | *p* | F | *p* | F | *p* | F | *p* |
| grassland – tree | 6.68 | **0.001** | 4.90 | **0.002** | 1.60 | 0.198 | 1.04 | 0.315 |
| grassland – edge | 7.22 | **0.001** | 5.40 | **0.002** | 8.04 | **0.002** | 10.68 | **0.002** |
| grassland – forest | 8.13 | **0.001** | 8.04 | **0.002** | 8.13 | **0.002** | 11.94 | **0.002** |
| tree – edge | 4.85 | **0.001** | 2.84 | **0.005** | 5.49 | **0.002** | 8.80 | **0.002** |
| tree – forest | 5.01 | **0.001** | 3.60 | **0.002** | 5.22 | **0.002** | 9.12 | **0.002** |
| edge – forest | 2.10 | **0.035** | 1.56 | 0.094 | 1.23 | 0.290 | 1.33 | 0.278 |

**Table S6.** Pairwise comparisons of community-weighted means (CWMs) in the four habitat types of wood-pastures with permutational multivariate analysis of variance (PERMANOVA). The *p*-values were adjusted with the FDR method. Significant differences (*p* < 0.05) are marked with boldface.

|  | PLANTS | | | | ANTS | | | |
| --- | --- | --- | --- | --- | --- | --- | --- | --- |
|  | Spring | | Summer | | Spring | | Summer | |
|  | F | *p* | F | *p* | F | *p* | F | *p* |
| grassland – tree | 10. 09 | **0.006** | 1.01 | 0.390 | 1.20 | 0.306 | 1.47 | 0.204 |
| grassland – edge | 9.78 | **0.003** | 8.69 | **0.002** | 8.64 | **0.003** | 12.77 | **0.003** |
| grassland – forest | 13.02 | **0.003** | 24.92 | **0.002** | 14.10 | **0.003** | 11.75 | **0.003** |
| tree – edge | 4.05 | **0.032** | 3.56 | **0.044** | 5.58 | **0.009** | 8.39 | **0.003** |
| tree – forest | 4.12 | **0.032** | 11.57 | **0.002** | 8.57 | **0.004** | 7.93 | **0.003** |
| edge – forest | 1.62 | 0.204 | 3.42 | 0.058 | 1.77 | 0.196 | 0.07 | 0.956 |

**Table S7.** Pearson correlation coefficients (r) of environmental variables with the first and second RLQ axes for plants and ants in both seasons according to the fourth-corner analyses. Significant associations (*p* < 0.05) are marked with boldface. *P*-values were adjusted for multiple comparisons using the FDR method.

| **PLANTS**  **SPRING** | Irradiation | | Air temperature | | Relative air humidity | | Soil temperature | | Soil moisture | | Bare ground | | Litter | |
| --- | --- | --- | --- | --- | --- | --- | --- | --- | --- | --- | --- | --- | --- | --- |
|  | test | *p* | test | *p* | test | *p* | test | *p* | test | *p* | test | *p* | test | *p* |
| Axis 1 | -0.35 | **0.006** | -0.03 | 0.881 | 0.05 | 0.831 | -0.26 | 0.095 | -0.26 | **0.012** | -0.07 | 0.726 | 0.34 | **0.004** |
| Axis 2 | -0.16 | 0.383 | -0.12 | 0.508 | 0.03 | 0.881 | -0.02 | 0.881 | -0.04 | 0.831 | 0.24 | **0.012** | -0.18 | 0.205 |
|  |  |  |  |  |  |  |  |  |  |  |  |  |  |  |
| **PLANTS**  **SUMMER** | Irradiation | | Air temperature | | Relative air humidity | | Soil temperature | | Soil moisture | | Bare ground | | Litter | |
|  | test | *p* | test | *p* | test | *p* | test | *p* | test | *p* | test | *p* | test | *p* |
| Axis 1 | -0.44 | **<0.001** | -0.43 | **<0.001** | 0.49 | **<0.001** | -0.39 | **<0.001** | 0.14 | 0.210 | 0.19 | 0.077 | 0.24 | **0.021** |
| Axis 2 | -0.05 | 0.688 | -0.03 | 0.751 | -0.08 | 0.594 | -0.06 | 0.665 | -0.24 | **0.005** | -0.11 | 0.215 | 0.15 | 0.097 |
|  |  |  |  |  |  |  |  |  |  |  |  |  |  |  |
| **ANTS**  **SPRING** | Irradiation | | Air temperature | | Relative air humidity | | Soil temperature | | Soil moisture | | Bare ground | | Litter | |
|  | test | *p* | test | *p* | test | *p* | test | *p* | test | *p* | test | *p* | test | *p* |
| Axis 1 | -0.32 | 0.419 | -0.20 | 0.429 | 0.24 | 0.429 | -0.34 | 0.419 | -0.36 | 0.419 | -0.14 | 0.429 | 0.37 | 0.429 |
| Axis 2 | -0.18 | 0.462 | 0.04 | 0.787 | -0.27 | 0.429 | 0.14 | 0.527 | 0.16 | 0.462 | -0.16 | 0.429 | 0.27 | 0.429 |
|  |  |  |  |  |  |  |  |  |  |  |  |  |  |  |
| **ANTS**  **SUMMER** | Irradiation | | Air temperature | | Relative air humidity | | Soil temperature | | Soil moisture | | Bare ground | | Litter | |
|  | test | *p* | test | *p* | test | *p* | test | *p* | test | *p* | test | *p* | test | *p* |
| Axis 1 | -0.26 | 0.392 | -0.38 | 0.150 | 0.33 | 0.150 | -0.33 | 0.186 | 0.07 | 0.729 | -0.14 | 0.278 | 0.22 | 0.413 |
| Axis 2 | -0.16 | 0.619 | 0.09 | 0.729 | -0.11 | 0.720 | 0.03 | 0.879 | -0.25 | 0.150 | -0.03 | 0.735 | 0.23 | 0.413 |

**Table S8.** Results of the fourth-corner analyses for bivariate associations between plant and ant functional traits and environmental variables. Correlations are based on D2 statistics. Significant associations (*p* < 0.05) are marked with boldface. *P*-values were adjusted for multiple comparisons using the FDR method.

| **PLANTS**  **SPRING** | Irradiation | | Air temperature | | Relative air humidity | | Soil temperature | | Soil moisture | | Bare ground | | Litter | |
| --- | --- | --- | --- | --- | --- | --- | --- | --- | --- | --- | --- | --- | --- | --- |
|  | test | *p* | test | *p* | test | *p* | test | *p* | test | *p* | test | *p* | test | *p* |
| Plant height | 0.04 | 0.872 | 0.08 | 0.750 | -0.02 | 0.939 | -0.06 | 0.844 | -0.03 | 0.864 | -0.18 | 0.141 | 0.13 | 0.555 |
| SLA | -0.19 | 0.448 | -0.06 | 0.844 | 0.02 | 0.934 | -0.06 | 0.844 | -0.06 | 0.767 | 0.01 | 0.934 | 0.13 | 0.546 |
| LA | -0.08 | 0.767 | 0.04 | 0.872 | 0.07 | 0.743 | -0.13 | 0.666 | -0.16 | 0.302 | -0.17 | 0.141 | 0.29 | 0.050 |
| Chamaephyte | -0.04 | 0.857 | 0.04 | 0.773 | -0.04 | 0.743 | -0.04 | 0.805 | 0.01 | 0.939 | -0.05 | 0.659 | 0.12 | 0.546 |
| Geophyte | -0.25 | 0.123 | -0.07 | 0.740 | 0.02 | 0.933 | -0.12 | 0.598 | -0.12 | 0.485 | 0.08 | 0.521 | 0.13 | 0.546 |
| Hemicryptophyte | 0.37 | **0.035** | 0.01 | 0.939 | 0.02 | 0.939 | 0.23 | 0.343 | 0.22 | 0.141 | -0.03 | 0.923 | -0.22 | 0.196 |
| Shrub | -0.08 | 0.721 | 0.01 | 0.950 | 0.03 | 0.773 | -0.10 | 0.573 | -0.09 | 0.574 | -0.07 | 0.533 | 0.15 | 0.444 |
| Tree | -0.15 | 0.485 | 0.07 | 0.721 | -0.04 | 0.780 | -0.14 | 0.485 | -0.12 | 0.444 | -0.14 | 0.269 | 0.24 | 0.097 |
| Therophyte | -0.11 | 0.682 | -0.02 | 0.939 | -0.01 | 0.950 | -0.03 | 0.934 | -0.07 | 0.740 | 0.15 | 0.229 | -0.16 | 0.444 |
| Flowering start | 0.29 | 0.097 | 0.12 | 0.666 | -0.09 | 0.696 | 0.20 | 0.444 | 0.20 | 0.118 | -0.10 | 0.464 | -0.07 | 0.773 |
|  |  |  |  |  |  |  |  |  |  |  |  |  |  |  |
| **PLANTS**  **SUMMER** | Irradiation | | Air temperature | | Relative air humidity | | Soil temperature | | Soil moisture | | Bare ground | | Litter | |
|  | test | *p* | test | *p* | test | *p* | test | *p* | test | *p* | test | *p* | test | *p* |
| Plant height | -0.26 | **0.047** | -0.27 | **0.034** | 0.31 | **0.010** | -0.24 | **0.047** | 0.15 | 0.119 | 0.15 | 0.127 | 0.12 | 0.162 |
| SLA | -0.08 | 0.641 | -0.06 | 0.738 | 0.12 | 0.459 | -0.04 | 0.846 | 0.15 | 0.090 | 0.11 | 0.303 | -0.03 | 0.796 |
| LA | -0.24 | 0.068 | -0.27 | **0.027** | 0.36 | **0.003** | -0.22 | 0.082 | 0.22 | **0.011** | 0.15 | 0.138 | 0.07 | 0.506 |
| Chamaephyte | -0.09 | 0.620 | -0.04 | 0.846 | 0.06 | 0.733 | -0.03 | 0.886 | 0.00 | 1.000 | 0.07 | 0.620 | 0.07 | 0.593 |
| Geophyte | -0.08 | 0.674 | -0.09 | 0.593 | 0.07 | 0.733 | -0.07 | 0.674 | 0.02 | 0.935 | -0.02 | 0.923 | 0.06 | 0.620 |
| Hemicryptophyte | 0.38 | **0.003** | 0.34 | **0.003** | -0.36 | **0.003** | 0.30 | **0.010** | -0.01 | 0.987 | -0.14 | 0.183 | -0.28 | **0.003** |
| Shrub | -0.21 | 0.068 | -0.21 | 0.087 | 0.17 | 0.178 | -0.18 | 0.134 | 0.00 | 0.987 | 0.01 | 0.987 | 0.16 | **0.043** |
| Tree | -0.31 | **0.003** | -0.27 | **0.027** | 0.32 | **0.003** | -0.26 | 0.053 | 0.00 | 0.987 | 0.14 | 0.175 | 0.22 | **0.017** |
| Therophyte | 0.05 | 0.790 | 0.06 | 0.738 | -0.03 | 0.846 | 0.06 | 0.738 | 0.00 | 0.987 | 0.06 | 0.699 | -0.04 | 0.725 |
| Flowering start | 0.22 | 0.090 | 0.23 | 0.058 | -0.19 | 0.145 | 0.24 | **0.047** | 0.00 | 0.998 | -0.07 | 0.593 | -0.11 | 0.205 |
|  |  |  |  |  |  |  |  |  |  |  |  |  |  |  |
| **ANTS**  **SPRING** | Irradiation | | Air temperature | | Relative air humidity | | Soil temperature | | Soil moisture | | Bare ground | | Litter | |
|  | test | *p* | test | *p* | test | *p* | test | *p* | test | *p* | test | *p* | test | *p* |
| Dominant | 0.16 | 0.819 | 0.01 | 0.990 | 0.01 | 0.978 | 0.12 | 0.819 | 0.08 | 0.869 | 0.24 | 0.819 | -0.33 | 0.819 |
| Intermediate | -0.04 | 0.965 | 0.10 | 0.819 | 0.06 | 0.917 | -0.11 | 0.819 | -0.10 | 0.819 | -0.07 | 0.819 | 0.16 | 0.819 |
| Subordinate | -0.14 | 0.819 | -0.05 | 0.917 | -0.04 | 0.924 | -0.06 | 0.917 | -0.03 | 0.960 | -0.20 | 0.819 | 0.25 | 0.819 |
| Cephalic size | 0.12 | 0.819 | -0.08 | 0.847 | 0.14 | 0.819 | -0.01 | 0.984 | -0.05 | 0.917 | -0.01 | 0.966 | -0.16 | 0.819 |
| Monogynous | 0.16 | 0.819 | 0.16 | 0.819 | -0.14 | 0.819 | 0.14 | 0.819 | 0.16 | 0.819 | 0.12 | 0.819 | -0.20 | 0.819 |
| Oligo-/polygynous | -0.16 | 0.819 | -0.16 | 0.819 | 0.14 | 0.819 | -0.14 | 0.819 | -0.16 | 0.819 | -0.12 | 0.819 | 0.20 | 0.819 |
| Temperature optimum | 0.30 | 0.819 | 0.20 | 0.819 | -0.24 | 0.819 | 0.29 | 0.819 | 0.34 | 0.819 | 0.15 | 0.819 | -0.34 | 0.819 |
| Humidity optimum | -0.07 | 0.917 | -0.11 | 0.819 | 0.21 | 0.819 | -0.21 | 0.819 | -0.22 | 0.819 | 0.06 | 0.909 | 0.07 | 0.924 |
| Eurytopic | -0.11 | 0.819 | -0.13 | 0.819 | 0.10 | 0.874 | -0.12 | 0.819 | -0.08 | 0.819 | -0.04 | 0.831 | 0.15 | 0.819 |
| Oligotopic | -0.16 | 0.819 | 0.02 | 0.960 | -0.27 | 0.819 | 0.18 | 0.819 | 0.16 | 0.819 | -0.07 | 0.831 | 0.17 | 0.819 |
| Polytopic | 0.18 | 0.819 | 0.03 | 0.960 | 0.25 | 0.819 | -0.17 | 0.819 | -0.17 | 0.819 | 0.10 | 0.819 | -0.23 | 0.819 |
| Stenotopic | 0.23 | 0.819 | 0.09 | 0.819 | -0.09 | 0.917 | 0.24 | 0.819 | 0.24 | 0.819 | -0.03 | 0.978 | -0.15 | 0.869 |
|  |  |  |  |  |  |  |  |  |  |  |  |  |  |  |
| **ANTS**  **SUMMER** | Irradiation | | Air temperature | | Relative air humidity | | Soil temperature | | Soil moisture | | Bare ground | | Litter | |
|  | test | *p* | test | *p* | test | *p* | test | *p* | test | *p* | test | *p* | test | *p* |
| Dominant | 0.23 | 0.809 | 0.17 | 0.809 | -0.14 | 0.809 | 0.18 | 0.809 | 0.03 | 0.964 | 0.01 | 0.993 | -0.15 | 0.809 |
| Intermediate | -0.22 | 0.809 | -0.26 | 0.809 | 0.29 | 0.809 | -0.23 | 0.809 | 0.07 | 0.809 | -0.02 | 0.937 | 0.14 | 0.809 |
| Subordinate | -0.07 | 0.903 | 0.02 | 0.993 | -0.06 | 0.903 | -0.01 | 0.993 | -0.08 | 0.809 | 0.01 | 0.993 | 0.05 | 0.986 |
| Cephalic size | -0.01 | 0.993 | -0.16 | 0.809 | 0.12 | 0.809 | -0.12 | 0.809 | 0.15 | 0.809 | -0.06 | 0.809 | -0.02 | 0.993 |
| Monogynous | 0.10 | 0.845 | 0.18 | 0.809 | -0.12 | 0.809 | 0.14 | 0.809 | -0.01 | 0.993 | 0.13 | 0.809 | -0.13 | 0.809 |
| Oligo-/polygynous | -0.10 | 0.845 | -0.18 | 0.809 | 0.12 | 0.809 | -0.14 | 0.809 | 0.01 | 0.993 | -0.13 | 0.809 | 0.13 | 0.809 |
| Temperature optimum | 0.28 | 0.809 | 0.35 | 0.809 | -0.30 | 0.809 | 0.30 | 0.809 | -0.01 | 0.993 | 0.09 | 0.809 | -0.20 | 0.809 |
| Humidity optimum | 0.02 | 0.993 | -0.17 | 0.809 | 0.16 | 0.809 | -0.14 | 0.809 | 0.10 | 0.809 | -0.21 | 0.809 | 0.12 | 0.809 |
| Eurytopic | -0.11 | 0.809 | -0.16 | 0.809 | 0.05 | 0.809 | -0.13 | 0.809 | -0.02 | 0.993 | -0.12 | 0.809 | 0.06 | 0.993 |
| Oligotopic | -0.11 | 0.844 | 0.11 | 0.809 | -0.07 | 0.874 | 0.04 | 0.986 | -0.17 | 0.809 | -0.05 | 0.809 | 0.23 | 0.809 |
| Polytopic | 0.12 | 0.809 | -0.08 | 0.874 | 0.11 | 0.809 | -0.04 | 0.986 | 0.21 | 0.809 | 0.10 | 0.809 | -0.22 | 0.809 |
| Stenotopic | 0.13 | 0.809 | 0.13 | 0.809 | -0.16 | 0.809 | 0.16 | 0.809 | -0.07 | 0.844 | 0.03 | 0.986 | -0.13 | 0.809 |

**Table S9.** Conditional R^2^ values for component models describing ant species richness and functional diversity. To incorporate vegetation metrics, we ran two sets of SEMs: 1) with plot-scale plant species richness (i.e., number of detected species) and functional diversity (expressed by Rao’s Quadratic Entropy values), 2) with plant composition (first ordination axis of a Principal Coordinate Analysis (PCoA) on the plant species-abundance matrix).

|  | **Plant metrics** | | | |
| --- | --- | --- | --- | --- |
|  | **Species richness and functional diversity** | | **Taxonomic composition (PCoA)** | |
|  | Spring | Summer | Spring | Summer |
| Ant species richness (SpR) | 0.18 | 0.12 | 0.17 | 0.04 |
| Ant functional diversity (FD) | 0.84 | 0.71 | 0.87 | 0.73 |
